# HIV-1 induced autophagy modulation in Langerhans cells

**DOI:** 10.1186/1742-4690-10-S1-P23

**Published:** 2013-09-19

**Authors:** Magdalena Czubala, John Paul Mitchell, Fabien Blanchet, Vincent Piguet

**Affiliations:** 1 Institute of Infection and Immunity, Cardiff University, Cardiff, UK

## Background and aim

Our research aims to decipher the mechanism and immune consequences imposed by HIV-mediated modulation of autophagy in challenged dendritic cell (DC) subsets, in particular Langerhans cells (LC). Autophagy is an ubiquitous cellular process involved in lysosomal-mediated degradation of host components as well as invading pathogens. In previous reports, HIV-1 was shown to modulate the autophagy flux in various immune cell types, including T cells, macrophages and myeloid dendritic cells. However, very little is known about the potential autophagy-driven antiviral activity in LC.

## Methods

LC used for this study were differentiated from either blood monocytes or the MUTZ-3 cell line model which phenotypically mimic LC morphology and immune features. LC were treated with wild type HIV-1, VSV-G pseudotyped HIV-1-derived lentivectors or recombinant HIV-1 envelopes. Levels of infection and cellular autophagy flux were analyzed by western blotting, FACS and confocal imaging.

## Results

We observe that, as previously described for myeloid DC, autophagy flux is compromised in LC after exposure to HIV-1 envelope or infectious virus. Hence, activation of the autophagy negative regulator, mTOR, is detected, and could account for the autophagy flux inhibition. Although we could confirm the HIV-1 restriction activity of langerin and SAMHD-1 in LC, it appears that shutting down autophagy flux is an early cellular target for the virus thus strongly suggesting that it might be involved in HIV-1 clearance shortly upon viral entry.

## Conclusion

In this study, we show that Langerin and SAMHD-1 are significantly restricting HIV-1 entry and replication in LC. However, an additional barrier to infection, such as autophagolysosome-mediated degradation of virus particles, may account for the low infection rate observed in LC. Our results suggest that autophagy flux is indeed dampened upon HIV-1 challenge which might favour virus progression. We further aim to examine the effect of autophagy modulation on virus infection of LC and consequent virus transmission to CD4^+^ T cells.

